# The Effects of *Elaeagnus angustifolia* L. on the Thyroid-Stimulating Hormone, Dehydroepiandrosterone-Sulfate, Prolactin and Cortisol Levels in Post-Menopausal Women: A Double-Blind, Randomized, and Placebo-Controlled Study

**DOI:** 10.3389/fphar.2021.654459

**Published:** 2021-07-07

**Authors:** Fatemeh Jalalvand, Arezou Rezaei, Bita Badehnoosh, Mehdi Yaseri, Mostafa Qorbani, Farzaneh Emaminia, Mahzad Shabani

**Affiliations:** ^1^ School of Biology, Damghan University, Damghan, Iran; ^2^ Institute of Biological Sciences, Damghan University, Damghan, Iran; ^3^ Department of Gynecology and Obstetrics, Faculty of Medical Science, Alborz University of Medical Science, Karaj, Iran; ^4^ Dietary supplement and Probiotic Research Center, Alborz University of Medical Science, Karaj, Iran; ^5^ Department of Epidemiology and Biostatistics, School of Public Health, Tehran University of Medical Sciences, Tehran, Iran; ^6^ Non-Communicable Diseases Research Center, Alborz University of Medical Sciences, Karaj, Iran; ^7^ Chronic Diseases Research Center, Endocrinology and Metabolism Population Sciences Institute, Endocrinology and Metabolism Research Institute, Tehran University of Medical Sciences, Tehran, Iran

**Keywords:** menopause, *Elaeagnus angustifolia* L., TSH, DHEA-S, prolactin, cortisol, traditional medicine, Iran

## Abstract

Menopause is the last menstrual period associated with a decline in ovarian steroid secretion and follicular loss. Hormone profile changes during menopause include a decrease in the production of estrogen, dehydroepiandrosterone (DHEA), and prolactin (PRL), and an increase in thyroid-stimulating hormone (TSH) and cortisol. Herbal medicines are considered as alternatives to hormone therapy. The studies on postmenopausal women have shown that *Elaeagnus angustifolia* L. (called “Senjed” in Persian) has some efficacy in improving sex hormone and lipid profiles, joint pain, and cardiovascular function, as the decrease in luteinizing hormone, low-density lipoprotein, and heart rate was significant. The aim of the present study was to evaluate the effects of *E. angustifolia* on TSH, DHEA-S, PRL, and cortisol levels and their ratios in postmenopausal women. It is assumed that the eventual effects of hormones on the brain and other tissues are determined by the balance between interdependent hormones. In the present randomized double-blinded placebo-controlled trial (https://en.irct.ir/search/result?query=IRCT20170227032795N4), fifty-eight postmenopausal women were randomly assigned to one of two medicinal herb (15 g of the whole *E. angustifolia* fruit powder) and placebo (7.5 g isomalt + 7.5 g cornstarch) groups. After 10 weeks of the treatment, the serum levels of TSH, DHEA-S, PRL, cortisol hormones, and their ratios were measured. The increase in the TSH, and cortisol levels, and cortisol/DHEA-S ratio and the decrease in prolactin and DHEA-S and the PRL/TSH, PRL/cortisol, and DHEA-S/TSH ratios after *E. angustifolia* consumption were significant only based on within-group but not on the between-group analysis. Based on between-group analyses, the changes in the hormone profile were not significant in the placebo group. According to Iranian tradition and folklore, *E. angustifolia* fruit is a symbol of female fertility. Therefore, its consumption is highly recommended to maintain health in the elderly, especially women. However, the observed outcomes about the effect of *E. angustifolia* on menopause were not completely in line with the Iranian folklore. *E. angustifolia* consumption did not significantly affect the hormone profile and ratios at the end of the ten-week trial, possibly due to the small sample size, short time, and the fact that our participants were postmenopausal women.

## Introduction

Menopause is one of the inevitable components of aging, including permanent menstrual cycles and loss of ovarian function. During this period, postmenopausal women experience decreased estrogen production in the ovary. Menopause occurs after 12 consecutive months of amenorrhea, indicating the end of fertility. Menopause typically occurs in midlife women, during their late 40s or early 50s, signaling the end of the fertile phase of a woman’s life (Davis et al., 2015).

Menopause symptoms can be divided into several categories: symptoms of the central nervous system, including hot flashes, sleep disorders, anxiety, depression, and migraines. Symptoms of the urogenital system are vaginal dryness and sexual dysfunction, such as decreased libido and dyspareunia. Postmenopausal women may also experience symptoms such as weight gain, osteoporosis, and joint pain (Monteleone et al., 2018; Wilhelms et al., 2019).

Hormones’ profile changes during menopause include the decrease in the production of dehydroepiandrosterone (DHEA) (Labrie et al., 2017) and prolactin (PRL) (Kwon et al., 2014) and an increase in thyroid-stimulating hormone (TSH, also known as thyrotropin) and cortisol (Pearce, 2007; Kalleinen et al., 2008). In females, estrogen is mostly synthesized by ovarian follicles (Merchenthaler, 2018). During menopause, ovarian function decreases, leading to decreased estrogen levels. DHEA is one of the main precursors of androgen that turns into estrogen and testosterone. DHEA-S, the sulfated form of DHEA, is the most abundant steroid hormone in serum (Scheffers et al., 2015). Estrogen stimulates the transcription of the PRL gene (Binart, 2017) and increases the serum levels of thyroxine-binding globulin (TBG), thereby reducing TSH. Consequently, in postmenopausal women, estrogen and TBG levels decrease; thus, TSH increases (Patisaul and Jefferson, 2010). It is assumed that the eventual effects of hormones on the brain and other tissues are determined by the balance between interdependent hormones (Maninger et al., 2009). In other words, hormone ratios are considered a straightforward way to analyze the effect of two independent hormones simultaneously (Sollberger et al., 2016). The changes in some hormone ratios in some diseases have been studied. For instance, some studies have shown that the PRL/cortisol ratio increased in autoimmune diseases such as systemic lupus erythematosus (Koeller et al., 2004), rheumatoid arthritis (Zoli et al., 2002), and Hashimoto’s disease (Legakis et al., 2001). However, to the best of our knowledge, few or no studies have been done on the ratios of TSH, DHEA-S, PRL, and cortisol in postmenopausal women.

There are various treatments for menopausal and postmenopausal women, including hormone replacement therapy (HRT), drug and non-drug therapies. Hormone replacement therapy includes the therapy with estrogen alone or estrogen combined with progesterone. Selective serotonin reuptake inhibitors (e.g., citalopram, paroxetine, and sertraline) and selective serotonin-norepinephrine reuptake inhibitors (e.g., venlafaxine and desvenlafaxine) are common types of postmenopausal drug therapies (Reviewed in Akhlaghi et al., 2015 and Yasui et al., 2003). Exercise therapy, meditation, yoga, aromatherapy, acupuncture, and behavior therapy are examples of non-drug therapies for menopause (Reviewed in Mintziori G et al., 2015). However, herbs have been a popular and well-known method of treating menopausal symptoms for centuries (Eden, J. A. 2012). Phytoestrogens are nonsteroidal, plant-derived compounds that are structurally or functionally similar to mammalian estrogens (Patisaul and Jefferson, 2010). Phytoestrogens are polyphenolic compounds and have antioxidant activity (Rodríguez-Landa et al., 2018). Phytoestrogens selectively bind to estrogen receptors, ERα and ERβ, like estrogens and are known to be effective in treating menopausal symptoms, such as osteoporosis, hot flashes and vasomotor symptoms, and anxiety and depression symptoms (Sirotkin and Harrath, 2014; Rodríguez-Landa et al., 2018).

*E. angustifolia L*. (Russian olive), known as Senjed in Persian, has high antioxidant activity and flavonoids in fruits (peel, flesh, and seeds) (Faramarz et al., 2015; Hamidpour et al., 2017). Senjed is used in Iranian traditional medicine (ITM) in the treatment of abdominal distension, diarrhea, jaundice and hemorrhoids, and for the heart and lungs, knee osteoarthritis, and joint pain (Mahboubi, 2018; Emaminia et al., 2020; Shabani et al., 2020).

In folkloric Iranian traditional medicine, *E. angustifolia* is recommended for the elimination of side effects of menopause. Desirable effects of *E. angustifolia* have been investigated in some cases such as wound repairing (Moezzi et al., 2009), the treatment of knee osteoarthritis (Ali Shiri et al., 2007), analgesic and anti-inflammatory effects in mice (Hosseinzadeh et al., 2003; Karimi et al., 2010), and symptomatic oral lichen planus (Taheri et al., 2010). The present study is a continuation of the previous works by Emaminia et al. (2020) and Shabani et al. (2020) and aims to investigate the effect of ripe *E. angustifolia* whole fruit on the serum level of TSH, DHEA-S, PRL, cortisol, and their ratios in postmenopausal women.

## Materials and Methods

### *Elaeagnus angustifolia* L. Preparation

*E. angustifolia* fruits were products of Damghan’s gardens (Semnan province, Iran) in October 2017. The *E. angustifolia* specimen was kept in the herbarium of Damghan university [Voucher number, Amirahmadi:1842 (DU000584)]. After the authors of this paper, Dr. Atefe Amirahmadi (Ph.D. of Plant Biosystematics, School of Biology, Damghan University) and Dr. Anna Abodolshahi (Ph.D. of Food science and technology (Food Safety Research Center, Semnan, Iran), confirmed the quality and health of the fruits, the whole fruit powder was prepared as previously described (Fantasia and Sutherland, 2014; Nikniaz et al., 2015). Isomalt from Puyakabak Manufacturing and Trading Company (Batch No 1604112, Tehran, Iran) and corn starch from the Bijan Pharmacy (Tehran, Iran) were also prepared with food grade. The powders were further sealed into packs separately, each pack containing 15 g of *E. angustifolia* whole fruit and isomalt + cornstarch powder (1:1 ratio).

Herbal medicine and placebo packaging were coordinated by a person who did not play a role in this project in two packages with different color schemes (red and blue, respectively). Supervisors and project students and those involved in clinical trials were unaware of the nature of these packages until the end of designing, receiving the latest test results, and analyzing the data.

**FIGURE 1:**
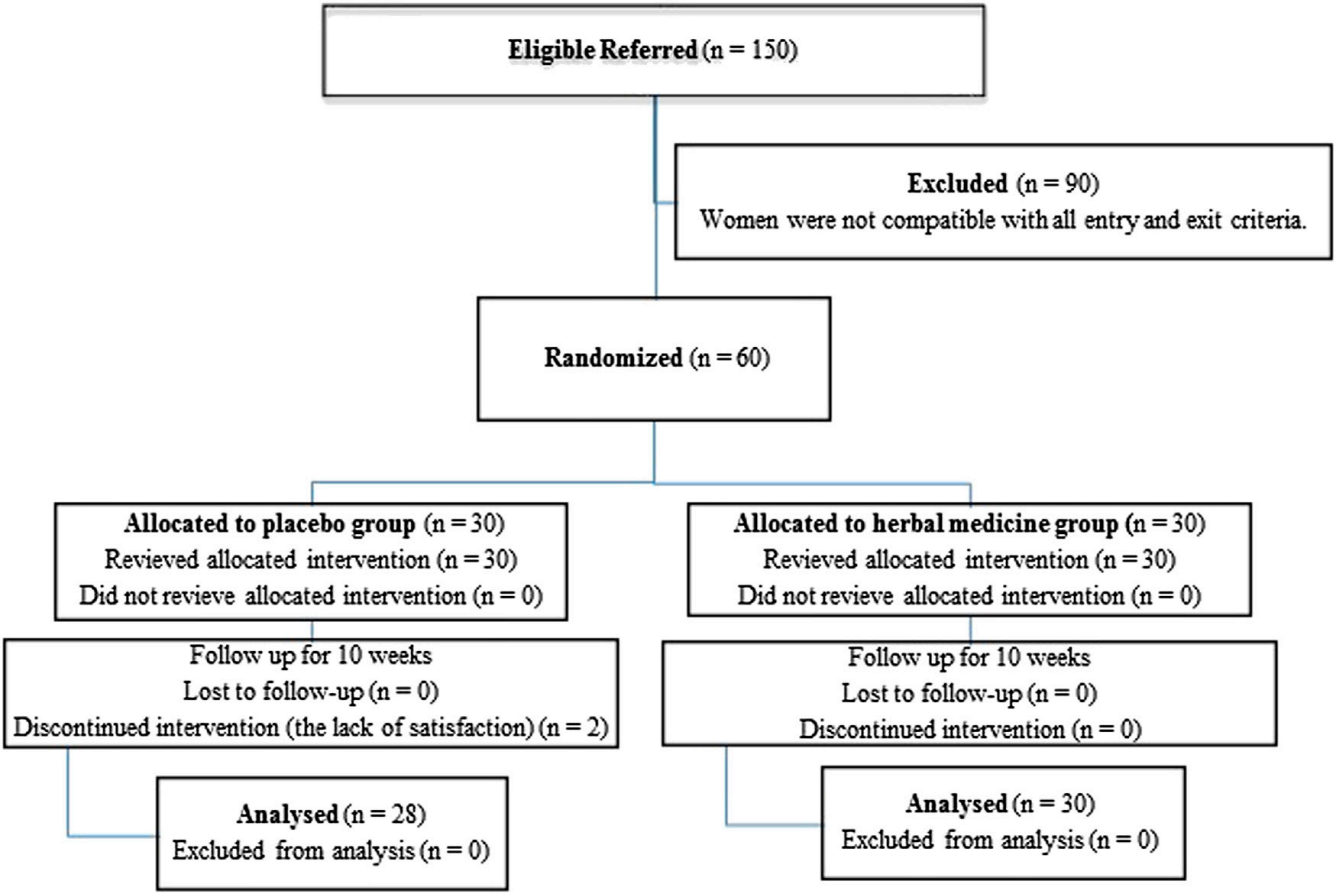
Flow chart for the participants’ enrollment, randomization, and retention. Participants in the herbal medicine and placebo groups received 15 g/day of *E. angustifolia* L. fruit powder and (7.5 g corn starch + 7.5 g isomalt)/day, respectively.

The blue (placebo) and red (herbal medicine) packages were stored in a refrigerator for food before delivery to the participants, and they were suggested to store their packages in the refrigerator before consumption. Figure 1 shows the flowchart of participant’s recruitment and retention.

**GRAPHICAL ABSTRACT:**
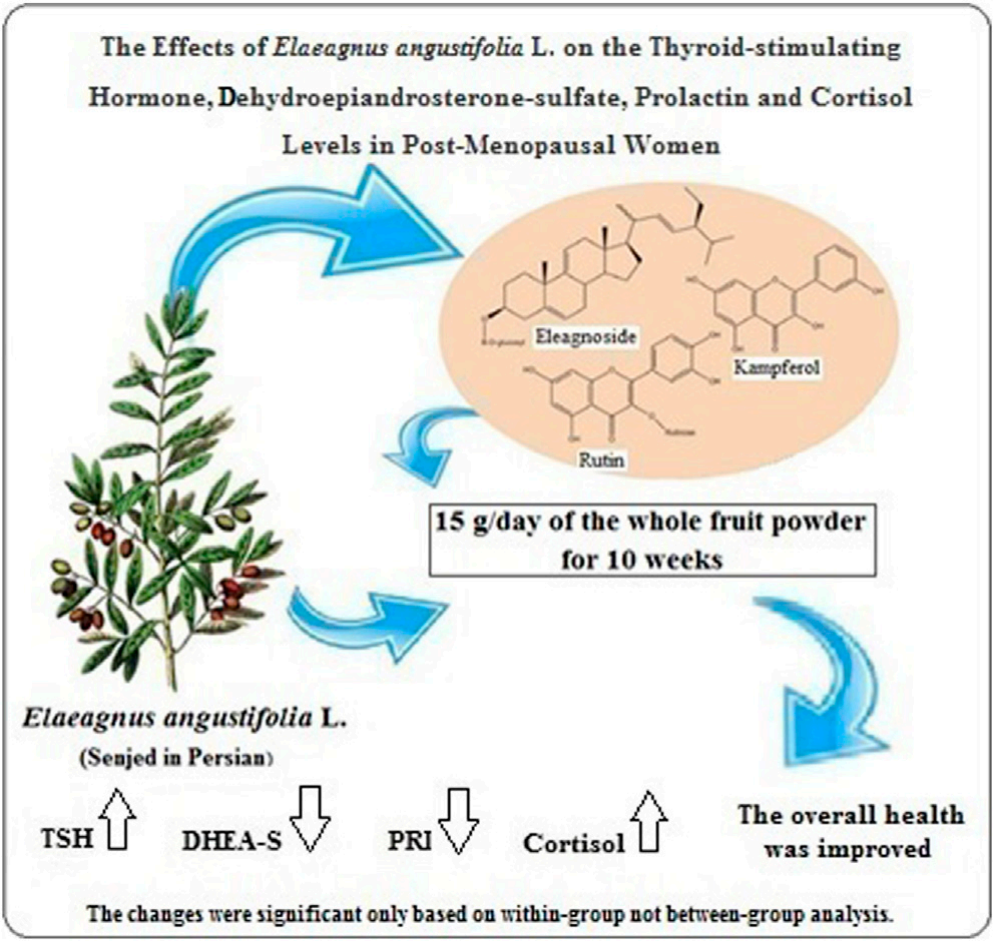
The effects of *Elaeagnus angustifolia* L. on the thyroid-stimulating hormone, dehydroepiandrosterone-sulfate, prolactin and cortisol levels in post-menopausal women. The changes were significant only based on within-group not between group analysis.

### Study Design

This study follows the CONSORT 2010 checklist of clinical trials (Schulz KF et al., 2010) and is a part of a double-blind, randomized placebo-controlled clinical trial with the ethical code Abzums.Rec.1396.162 and registration code IRCT20170227032795N4 in the clinical trial system. Among the postmenopausal women referring to the gynecology clinic of the Kamali hospital (Karaj, Alborz, Iran), 60 postmenopausal women were invited after gynecologist confirmation, personal interview, explanation of the purpose and method of the study, and receiving informed written consent.

### Subject Selection

#### Inclusion Criteria

The postmenopausal women were invited to participate in the study based on inclusion and exclusion criteria. The inclusion criteria included menopausal women aged 40–70 years and serum cholesterol levels between 200 and 300 mg/ml. In the initial assessment, blood pressure, heart rate, total cholesterol, and FSH levels were evaluated to confirm being menopause. Women who were included in the study did not have a risk factor for not using medication to reduce their lipid level or did not have any history of receiving hormone injections in the last 6 months, and also had no history of daily intake or allergy to *E. angustifolia* (Emaminia et al., 2020; Shabani et al., 2020).

#### Exclusion Criteria

Exclusion criteria included metabolic disorders such as diabetes, cardiovascular and renal diseases, consuming alcohol, and psychiatric drugs, smoking cigarette, and hookah **(** Emaminia et al., 2020; Shabani et al., 2020).

### Randomization and Experimental Groups

A simple random selection method was used for assigning the postmenopausal women into two control and intervention groups, each with 30 participants. A person who did not participate in this trial did this randomization. The numbers 1–60 were written on paper of the same color and shape. The paper was put in a pot after folding. Then, the bowl components were stirred. The paper was taken randomly by a person who did not have a role in this project. The first 30 numbers that came out of the bowl were intended for people who were supposed to be in the blue group. The 30 numbers remaining in the bowl were considered as the red group. Each study group took 15 g of herbal medicine (whole *E. angustifolia* fruit powder) or placebo (a combination of 7.5 g of corn starch and 7.5 g of isomalt) equally for 10 weeks every day. The dosage and duration of Senjed and placebo consumption were selected based on studies on the effect of this fruit on osteoarthritis and the lipid profile of obese women (Ali Shiri et al., 2007; Moezzi et al., 2009), and the ineffectiveness of cornstarch and isomalt on blood glucose and lipid profiles (Karimi et al., 2010; Ebrahimi et al., 2014; Nikniaz et al., 2015).

Participants were recommended to eat the packages’ contents after breakfast and with milk, if possible (Nikniaz et al., 2016). Two people involved in this clinical trial, Farzaneh Emaminia and Mahzad Shabani, were in touch with participants twice a week regularly to record the participants’ general condition, satisfaction, or dissatisfaction.

### Data Collection and Sampling

For data collection, fasting blood samples were taken, and an interview questionnaire was filled, which was done in Iranzamin Lab (Karaj) in two steps, including at the beginning and the end of the trial after 10 weeks of consumption of herbal medicine/placebo and the ending stage.

### Hormone Profile

Blood analysis was done for four hormones, including TSH, DHEA-S, PRL, and cortisol. To measure hormones, the serum sample was prepared from 3 ml of patients’ fasting blood samples. Enzyme-Linked Immunosorbent Assay (ELISA) and commercial kits of Bio Karpira Company (Iran) were used to measure the PRL and TSH hormones. An automated Immulite 2000 (Siemens, United States) working on the Chemiluminescence technique was used to measure DHEA-S and cortisol hormones.

### Statistical Analysis

Statistical analysis was performed using SPSS software version 25. In the initial analysis, the normality of data distribution was assessed with the Shapiro-Wilk test. The groups were compared with Independent Samples *t*-test if the parameter distribution was normal, and for the parameter with abnormal distribution, the Mann-Whitney test was used. The within-group comparisons were performed using a paired sample *t*-test or Wilcoxon test for data with the normal or abnormal distribution. The *p*-values of within-group analyses indicate the importance of the difference in the studied parameters in each research group of herbal medicine or placebo before and after the trial period. Between-group *p*-values indicate the importance of the difference in the studied parameters between herbal medicine and placebo groups before and after the trial. Change score *p*-value was used to compare between-group *p*-values of each studied parameter. A *p*-value < 0.05 was considered statistically significant. Since we had several outcomes, all *p*-values were adjusted using the Benjamini-Hochberg correction method to control the multiple comparisons problem (Benjamini et al., 1995).

Based on the central limit theorem (CLT) (Kwak and Kim, 2017), the sample means that the probability distribution of the studied characteristics in a sample size of 30 is close to the normal probability distribution. The sample size was also calculated based on the difference in (pre-post) change of TSH, DHEA-S, and PRL that have been considered main outcomes. Among these items, the change in TSH provided the largest sample. The final sample size was calculated based on this parameter. To have a power of 80% (type II error of 20%) and type I error of 5% to detect a difference of changes as big as 2, when the standard deviation of TSH was believed to be 2.7 in each group, a sample size of 29 was needed (Table 1). We included 30 samples in each group to compensate for the probable missing.

**TABLE 1:** Calculation of the sample size based on the difference in change (pre-post) of the levels of thyroid-stimulating hormone (TSH), dehydroepiandrosterone-sulfate (DHEA-S), prolactin (PRL).

Parameter	SD	Difference	Power	Sample size
TSH	2.7	2	80	29
DHEA-S	40	30	80	28
PRL	180	150	80	23

## Results

In this clinical trial, the parameters studied in postmenopausal women in both herbal medicine and placebo groups were measured before treatment and at the end of the trial. In the placebo group, two menopausal women refused to continue their cooperation. Therefore, data were reported for 58 postmenopausal women in two groups after 10 weeks of treatment. In both herbal medicine (*E. angustifolia*) and placebo (isomalt and cornstarch) groups, the women had a normal distribution based on age, as previously reported (Emaminia et al., 2020; Shabani et al., 2020). As shown in Table 2, all 58 participants’ average age was 55.39 years; which was 56.63 years in the herbal medicine group (*n* = 30) and 54.07 years in the placebo group (*n* = 28), respectively. The difference between the two study groups was not significant in this regard (*p* = 0.121). There was no statistically significant difference between the two herbal medicine and placebo groups in terms of baseline characteristics such as education, job, type of delivery, lactation period, exercise, consumption of any booster pill, harmful habits, and misadventure events in the past 5 years (Table 2).

**TABLE 2:** Comparison of the baseline characteristics of participants between two study groups.

Variable	Category	Groups		Total (*n* = 58)	*p*-value
		Herbal medicine (*n* = 30)	Placebo (*n* = 28)		
Age	Numerical	56.63 ± 5.43	54.07 ± 6.90	55.39 ± 6.26	0.121^t^

Variables	Category	Total	Groups				Total		*p*-value^*^
			Herbal medicine		Placebo				
			Num	Percentage	Num	Percentage	Num	Percentage	
Education	Uneducated	1	4	13.3%	2	7.1%	6	10.3%	0.677^tt^
	1–6 years	2	16	53.3%	17	60.7%	33	56.9%	
	6–12 years	3	8	26.7%	5	17.9%	13	22.4%	
	12+	4	2	6.7%	4	14.3%	6	10.3%	
Job	Yes	1	2	6.7%	4	14.3%	6	10.3%	0.415^**^
	No	2	28	93.3%	24	85.7%	52	89.7%	
Type of delivery	Natural childbirth	1	23	76.7%	23	60.7%	40	69.0%	0.265^tt^
	Cesarean delivery	2	2	6.7%	7	25.0%	9	15.5%	
	Both	3	5	16.7%	2	7.1%	7	12.1%	
	None of them	4	0	0.0%	2	7.1%	2	3.4%	
Lactation period	No	1	0	0.0%	3	10.7%	3	5.2%	0.466^tt^
	Less than 6 months	2	2	6.7%	3	10.7%	5	8.6%	
	Less than 1 year	3	3	10.0%	0	0.0%	3	5.2%	
	More than 1 year	4	25	83.3%	22	78.6%	47	81.0%	
Exercise	No	1	14	46.7%	13	46.4%	27	46.6%	0.280^tt^
	Every week	2	6	20.0%	1	3.6%	7	12.1%	
	Twice in week	3	6	20.0%	3	10.7%	9	15.5%	
	Every day	4	4	13.3%	11	39.3%	15	25.9%	
Consumption of any type of booster pill	Yes	1	17	56.7%	12	42.9%	29	50.0%	0.293^*^
	No	2	13	43.3%	16	57.1%	29	50.0%	
Harmful habits	No	1	27	90.0%	26	92.9%	53	91.4%	0.701^tt^
	Cigarette	2	1	3.3%	0	0.0%	1	1.7%	
	Hookah	3	1	3.3%	2	7.1%	3	5.2%	
	Alcoholic drinks	4	1	3.3%	0	0.0%	1	1.7%	
Misadventure event over 5 years ago	Yes	1	17	56.7%	18	64.3%	35	60.3%	0.553^*^
	No	2	13	43.3%	10	35.7%	23	39.7%	

N, the number of participants; t: *t* test, tt: Mann Whitney test, *, Chi-Square test, **, Fisher’s test. A *p*-value < 0.05 was considered statistically significant.

Table 3 shows the results of the effects of herbal medicine and placebo treatment on the studied hormones. Considering within-group *p*-values, the TSH amount showed a significant increase in the herbal medicine (*p* = 0.007) group after 10 weeks of treatment, but this change in the placebo group was not significant. By assessing the changes between the two study groups, the changes in the amount of TSH were not significant before and after treatment (change score *p*-value = 0.857).

**TABLE 3:** Comparison of the levels of thyroid-stimulating hormone (TSH), dehydroepiandrosterone-sulfate (DHEA-S), prolactin (PRL), and cortisol and their ratios between the two study groups.

a) Parameters with non-normal distribution. Data are shown as median ± interquartile range (IQR)				
Variables (unite)	Time	Herbal medicine groups (*n* = 30)	Placebo groups (*n* = 28)	*p-value*
		Median ± IQR	Median ± IQR	
**TSH (µIU/ml)**	Pre-treatment	2.51 ± 2.12	2.12 ± 1.77	0.597^tt^
	Post-treatment	2.93 ± 2.90	2.61 ± 1.55	0.780^tt^
	**Change score**	0.63 ± 2.33	0.04 ± 2.62	0.857^tt^
	Within groups	**0.007** ^**w**^	0.374^w^	—
**DHEA-S (µg/ml)**	Pre-treatment	52.30 ± 42.20	36.20 ± 37.72	0.486^tt^
	Post-treatment	17.85 ± 33.85	19.50 ± 36.41	0.950^tt^
	**Change score**	−32.26 ± 48.49	−14.73 ± 38.92	0.857^tt^
	Within groups	**<0.001** ^**w**^	**0.015** ^**w**^	—
**PRL (mIU/L)**	Pre-treatment	193.10 ± 98.33	191.41 ± 114.66	0.656^tt^
	Post-treatment	161.50 ± 145.06	159.76 ± 221.52	0.905^tt^
	**Change score**	−53.00 ± 177.23	−68.55 ± 187.95	0.938^tt^
	Within groups	**0.015** ^**w**^	**0.063** ^**w**^	—
**PRL/TSH**	Pre-treatment	73.21 ± 105.38	99.74 ± 85.79	0.656^tt^
	Post-treatment	29.17 ± 61.02	43.56 ± 100.54	0.905^tt^
	**Change score**	−31.11 ± 105.46	−41.14 ± 114.23	0.938^tt^
	Within groups	**0.007** ^**w**^	**0.015** ^**w**^	—
**PRL/DHEA-S**	Pre-treatment	3.99 ± 5.16	4.89 ± 5.76	0.597^tt^
	Post-treatment	7.62 ± 180.80	7.79 ± 1,323.56	0.905^tt^
	**Change score**	2.77 ± 182.55	3.10 ± 1,324.24	0.938^tt^
	Within groups	0.070^w^	0.084^w^	—
**PRL/Cortisol**	Pre-treatment	31.62 ± 26.77	18.76 ± 13.10	0.155^tt^
	Post-treatment	12.67 ± 14.79	9.91 ± 15.58	0.780^tt^
	**Change score**	−15.16 ± 30.60	−8.21 ± 26.64	0.857^tt^
	Within groups	**<0.001** ^**w**^	**0.034** ^**w**^	—
**Cortisol/DHEA-S**	Pre-treatment	0.15 ± 0.24	0.29 ± 0.23	0.145^tt^
	Post-treatment	0.56 ± 20.31	0.63.±95.29	0.605^tt^
	**Change score**	0.35 ± 20.48	0.32 ± 95.41	0.938^tt^
	Within groups	**<0.001** ^**w**^	**0.015** ^**w**^	—
**DHEA-S/TSH**	Pre-treatment	19.77 ± 32.16	18.76 ± 23.31	0785^tt^
	Post-treatment	5.15 ± 9.21	6.77 ± 15.25	0.905^tt^
	**Change score**	−13.30 ± 28.60	−7.73 ± 22.95	0.857^tt^
	Within groups	**<0.001** ^**w**^	**0.015** ^**w**^	—
**Cortisol/TSH**	Pre-treatment	2.91 ± 2.93	4.33 ± 6.02	0.153^tt^
	Post-treatment	3.49 ± 3.70	5.06 ± 5.61	0.780^tt^
	**Change score**	0.18 ± 2.87	1.06 ± 6.37	0.938^tt^
	Within groups	0.586^w^	0.767^w^	—

b) Parameters with the normal distribution. Data are shown as mean ± standard deviation							
Variables (unite)	Time	Herbal medicine groups (*n* = 30)	Placebo groups (*n* = 28)	Mean difference	95% Confidence interval of the difference		*p-value*
		Mean ± SD	Mean ± SD		Lower	Upper	
**Cortisol (µg/dl)**	Pre-treatment	7.82 ± 3.70	10.77 ± 5.76	−2.957	−5.489	−0.425	0.145^t^
	Post-treatment	11.42 ± 5.12	13.28 ± 6.06	−1.862	−4.810	1.086	0.780
	**Change score**	3.60 ± 6.02	2.51 ± 7.99	1.095	−2.613	4.804	0.938
	Within groups	**0.006** ^**p**^	0.135^p^	—	—	—	—

Between-group *p*-values show the significance of differences in the studied parameters between two study groups before and after the trial period. Within-group *p*-values show the significance of differences in the studied parameters of each study group before and after the trial period. Change score *p*-values compare between *p*-values of each studied parameter. t, T-test; tt, Mann-Whitney test; w, Wilcoxon test; *p*, Pair t-test. Bold figures show that the difference is significant (*p* < 0.05).

The DHEA-S amount showed a significant decrease in both herbal medicine and placebo groups after ten-week treatment (within-group *p*-values < 0.001 and = 0.015, respectively). However, this hormone’s changes were not significant before and after treatment between the two study groups (change score *p*-value = 0.857).

The herbal medicine group’s increase in cortisol levels was significant, but not in the placebo group (within-group *p*-values = 0.006 and 0.135, respectively). The cortisol changes were not significant between the herbal medicine and placebo groups at the end of the trial (change score *p*-value = 0.938).

The decrease in PRL levels was significant only in herbal medicine group but not in placebo group after ten-week treatment (within-group *p*-values = 0.015 and 0.063, respectively). However, the hormone level changes were not significant between the two study groups at the end of treatments (change score *p*-value = 0.938).

For hormone ratios, considering within-group *p*-values, the PRL to TSH ratio showed a significant decrease in both herbal medicine (*p* = 0.007) and placebo (*p* = 0.015) groups at the end of the trial. However, assessing the change score *p*-values showed that the decrease in this ratio was not significant between the two study groups (*p* = 0.938).

The increase in the ratio of PRL to DHEA-S was not significant in herbal medicine and placebo groups (within-group *p*-values = 0.070 and 0.084, respectively). The change in this ratio was not significant between the two study groups (change score *p*-value = 0.938) after ten-week treatment.

In within-group *p*-value analysis, PRL to cortisol ratio showed a significant decrease in both herbal medicine (*p* < 0.001) and placebo (*p* = 0.034) groups at the end of the trial. However, the decrease in this ratio was not significant between the two study groups (change score *p*-value = 0.857).

The increase in the cortisol to DHEA-S ratio was significant in both herbal medicine and placebo groups after ten-week treatment (within-group *p*-values <0.001 and 0.015, respectively). The changes in this ratio were not significant between the two study groups (change score *p*-value = 0.938).

The decrease in the ratio of DHEA-S to TSH was significant in the herbal medicine and placebo groups (within-group *p*-values < 0.001 and 0.015, respectively), but not between two study groups (change score *p*-value = 0.857).

Considering within-group *p*-values, cortisol to TSH ratio did not change significantly in both herbal medicine (*p* = 0.586) and placebo (*p* = 0.767) groups. Also, the change in this ratio was not significant between the two study groups at the end of the trial (*p* = 0.938).

## Discussion

Menopause is a stage in women’s life that affects their quality of life. To treat menopausal symptoms, lifestyle changes or hormonal and non-hormonal therapies are suggested, each with its benefits and risks. Hormone therapy can lead to certain diseases, such as cancer (Fantasia and Sutherland, 2014). Accordingly, women turn to complementary and alternative therapies (Dietz et al., 2016). In Iranian folklore medicine, oral intake of *E. angustifolia* fruit powder is considered useful for osteoarthritis, enuresis, liver, stomach, intestine, heart and lungs wounds and asthma (Reviewed in Mahboubi, 2018). Furthermore, according to Iranian tradition and folklore, *E. angustifolia* fruit symbolizes love, happiness, wisdom, and female fertility. The exocarp, endocarp, and seed of *E. angustifolia* fruit are recommended to strengthen skin, muscle, and skeletal function, respectively. Accordingly, its consumption is highly recommended to maintain health in elderly, especially women. In the present study and the continuation of our previous studies (Emaminia et al., 2020; Shabani et al., 2020), the effect of *E. angustifolia* on some serum hormones was studied in postmenopausal women for the first time.

In our previous study, i.e., the study by Emaminia et al. (2020), we showed that the increase in FSH and estradiol levels, and the decrease in progesterone levels were not significant after *E. angustifolia* consumption. However, the LH level decreased significantly. Moreover, the improvement in joint pain was significant in postmenopausal participants. In our another study, i.e., the study by Shabani et al. (2020), we showed that a ten-week treatment with *E. angustifolia* significantly lowered LDL-C level and heart rate in postmenopausal participants without significant change in the glycemic profile.

According to between-group analyses, in the current study, after 10 weeks of treatment with *E. angustifolia*, no significant changes were observed in the studied parameters. However, in the within-group analyses, the levels of TSH, cortisol, and cortisol/DHEA-S increased significantly after taking *E. angustifolia*. At the same time, there was a significant decrease in the levels of DHEA-S, PRL, PRL/TSH, PRL/cortisol, and DHEA-S/TSH at the end of the trial. The changes in the ratios of PRL/DHEA-S and cortisol/TSH were not significant.

High TSH levels can have adverse effects on cardiac function. Decreased thyroid hormones (T3 and T4) can lead to increased atherogenic markers total cholesterol (TC) and low-density lipoprotein cholesterol (LDL-C) (Wanjia et al., 2012). TSH level may increase with age (Bremner et al., 2012; Leng and Razvi, 2019). Due to the low individuality index of TSH, it is not possible to determine an accurate TSH upper limit at the individual level from population data. With the current upper limit of the serum TSH reference range in the elderly, overdiagnosis of hypothyroidism is possible (Leng and Razvi, 2019). In addition to levels of thyroid hormones, particularly free T4, other studies are needed to define an age-specific TSH reference range as part of clinical practice (Bremner et al., 2012; Leng and Razvi, 2019). In the current study, the levels of thyroid hormones were not measured. However, based on our previous study, it can be concluded that the insignificant 16.73% increase in the TSH level from 2.51 μIU/ml before treatment to 2.93 μIU/ml at the end of the trial had no negative impact on the lipid profile and cardiovascular function in postmenopausal participants. This is because treatment with *E. angustifolia* had no significant effect on TC and triglyceride (TG) levels, systolic and diastolic blood pressure, but significantly decreased heart rate and LDL-C (Shabani et al., 2020).

In the current study, the increase in cortisol levels and the decrease in DHEA-S levels were significant only in within-group but not between-group comparisons. The cortisol/DHEA-S ratio also increased significantly in the herbal medicine and placebo groups only in within-group but not between-group analyses (Table 3).

DHEA is a multifunctional hormone with immune enhancing, breast-cancer preventive, and antiaging effects (Yen et al., 1998; Ferrari et al., 2001a; b). DHEA is a precursor to androgens and estrogen and its conversion into estrone is the major source of estrogen in postmenopausal women (Rodwell et al., 2018). DHEA-S is the most abundant steroid hormone in serum (Ruan et al., 2017) and can modulate lipid and glucose metabolism (Yen and Laughlin, 1998). As DHEA-S levels decrease, pain threshold and tolerance decrease (De Abreu Freitas., 2012). However, despite these changes, postmenopausal women reported a reduction in pain after *E. angustifolia* consumption (Emaminia et al., 2020).

In our previous studies, i.e., the study by Emaminia et al. (2020), we showed that the estradiol (E2) and testosterone (T) levels increased insignificantly by 28.29 and 59.59%, respectively, after treatment with *E. angustifolia*. (Emaminia et al., 2020). Furthermore, a calculation using the current data and the result obtained from the study by Emaminia et al. (2020) showed a decrease in the ratios of DHEA-S/E2 (65.10%) and DHEA-S/P (47.35%), and DHEA-S/T (54.53%), and an increase in E2/T (26.45%) and E2/P (13.81%) ratios. In the current study, the DHEA levels were not measured. However, the 65.86% obvious reduction in DHEA-S in the herbal medicine group could be due to the conversion of DHEA to progesterone, testosterone, estradiol, and oxygenated DHEA metabolites rather than conversion into DHEA-S in the peripheral tissues (El Kihel et al., 2012; Rodwell et al., 2018) which requires the assessment of key enzymes in these pathways as well as other DHEA metabolites.

In our previous study, a 45.98% decrease in progesterone hormone (P) level was seen after treatment with herbal medicine which was significant in within-group (*p* = 0.049), but not in between-group (change score *p*-value = 0.273) analyses (Emaminia et al., 2020). One of the reasons for the increase in cortisol in the current study might be the conversion of progesterone into cortisol, which requires the assessment of related enzymes in this pathway. It is noteworthy to mention that the level of TC did not change significantly after ten-week treatment with *E. angustifolia* as shown in our previous study, i.e., the study by Shabani et al. (2020).

Cortisol increases lipolysis, which actually breaks down triglycerides into glycerol and fatty acid. It also reduces glucose consumption and insulin sensitivity (PNandhini et al., 2019). In the previous study, TG levels decreased after *E. angustifolia* treatment, although this change was not significant (change score *p*-values = 0.065). But, fasting blood glucose (within-groups *p*-value = 0.002, change score *p*-value = 0.303) and insulin (change score *p*-value = 0.04) increased; however, both remained within normal range (Shabani et al., 2020). These glycemic profile changes due to herbal medicine consumption can be related to the effect of herbal medicine on cortisol. Understanding the exact mechanism of these changes in the hormonal and glycemic profile of postmenopausal women requires further studies.

The relationship between cortisol and DHEA-S is usually considered in tandem. These two critical adrenal steroids have opposite effects on the central nervous system (Yen and Laughlin, 1998; Ferrari, et al., 2001a; b). The neurosteroid DHEA-S has anti-glucocorticoid, antihypertensive, anti-inflammatory, and neuroprotective effects (Maninger et al., 2009). Although cortisol secretion is generally well maintained with age, DHEA-S levels progressively decrease with aging. The molar ratio between cortisol and DHEA-S also increases with age (Yen and Laughlin, 1998; Ferrari et al., 2001b).

The treatment with both *E. angustifolia* and placebo increased the cortisol/DHEA-S ratio which was only significant in the within-group but not between-group analyses (Table 3). High serum cortisol/DHEA-S ratio (≥0.2 μg/dl) is a risk factor for sarcopenia in elderly diabetic patients. Chronic stress often increases cortisol secretion and decreases DHEA-S secretion (Phillips et al., 2010). Therefore, the ratio of DHEA-S/cortisol decreases (Yanagita et al., 2019). A decrease in DHEA-S levels and an increase in cortisol/DHEA-S ratio have been suggested as a possible mechanism in cancer-related mortality (Phillips et al., 2010). An increased cortisol/DHEA-S ratio was also shown in schizophrenia (Markopoulou et al., 2009) and treatment-resistant depression (Ritsner, 2009). DHEA and DHEA-S levels increase significantly in response to acute psychosocial stress along with significantly elevated cortisol, heart rate, and systolic and diastolic blood pressure in both men and women. However, the ability to increase these levels during acute psychosocial stress decreases with age. The molar ratio between cortisol and DHEA-S increases with stress (Lennartsson et al., 2012). The postmenopausal women in the present study did not suffer from specific metabolic diseases such as diabetes or neurodegenerative diseases such as schizophrenia. Furthermore, we showed previously that ten-week consumption of *E. angustifolia* reduced significantly heart rate without any significant change in blood pressure (Shabani et al., 2020). Accordingly, it can be concluded that the decreased DHEA-S level and increased cortisol/DHEA-S ratio, which were not significant, did not negatively affect on the participants and did not indicate an increased stress in them.

Postmenopausal women are at greater risk of developing atherosclerosis and hypertension. Evidence has shown that high levels of PRL may accelerate vascular aging in menopause and play a key role in increasing the prevalence of hypertension after menopausal transition (Amirzadegan et al., 2019). There has also been a relationship between high plasma PRL and cardiovascular mortality (Byberg et al., 2019). Anxiety is also associated with high levels of PRL (Barry et al., 2015). The amount of PRL in early postmenopausal women is different from that in the late postmenopausal women (Grattan and LeTissier, 2015). Ten weeks of *E. angustifolia* treatment caused a 16.36% decrease in PRL, which was only significant in within-group but not between-group comparisons (Table 3). Regarding the impact of hyperprolactinemia on cardiovascular function, the beneficial effects of *E. angustifolia* on heart rate and LDL-C level (Shabani et al., 2020) might be attributed to the decreased PRL level after *E. angustifolia* consumption.

The ratio of PRL/TSH in herbal medicine and placebo groups significantly decreased only in within-group but not between-group comparisons (Table 3). Studies suggest that pituitary hormones such as growth hormone, FSH, TSH, and PRL have important roles in regulating bone. Osteoporosis is also associated with thyroid dysfunction in older women. Fracture risk has been reported to be associated with low serum TSH levels (Colaianni et al., 2013). In hypothyroidism, the hypothalamus increases TSH production in the pituitary gland by producing thyrotropin-releasing hormone (TRH), which is required for normal physiological elevation of thyroxine levels. Prolonged hypothyroidism causes hyperplasia of pituitary thyrotropic cells. TRH has a poor excitatory effect on pituitary lactotrophic cells, which might result in a slight to moderate increase in PRL. In other words, in hypothyroidism, the concentrations of both TSH and PRL increases. In a report of a 67-year-old woman with a large pituitary mass resulting in a very high level of TSH, the PRL level also showed a significant increase (Ansari and Almalki, 2016). In postmenopausal women, plasma PRL levels decrease in parallel with the decrease in plasma estradiol. So, postmenopausal women have lower baseline plasma PRL levels than premenopausal women have (Markianos et al., 1996; Pałubska et al., 2017).

As far as we know, no studies have been done on the ratio of TSH and PRL in postmenopausal women. In the present study, the increase in TSH level and the decrease in PRL and also PRL to TSH ratio in herbal medicine group were only significant in the within-group analysis but not between two study groups at the end of the trial (Table 3). PRL deficiency reduces the risk of cardiovascular disease (Byberg et al., 2019). Increased TSH levels also lead to hypercholesterolemia and hypertriglyceridemia (Wanjia et al., 2012). Although the decrease in PRL was not significant after treatment with herbal medicine, we previously showed that *E. angustifolia* treatment also significantly reduced LDL-C and improved cardiovascular function and lipid profile in postmenopausal women (Shabani et al., 2020). Therefore, it can be concluded that the overall effect of changes in the two hormones TSH and PRL after treatment with herbal medicine on the lipid profile and cardiovascular function was positive.

In both within-and between-group analyses, PRL/DHEA-S hormonal ratio changes were not significant in the two study groups (Table 3). In studies on the relationship between hormones and sexual function, and the relationship between sex hormones and the quality of life, DHEA was the only hormone that had a significant negative relationship with sexual function, which was inversely related to sexual desire levels (Peixoto et al., 2019). Given that DHEA is an indirect testosterone precursor, the relationship between this hormone and sexual desire is expected to be positive. However, in the study mentioned above, higher DHEA levels were associated with lower sexual desire (Peixoto et al., 2019). It has been suggested that because DHEA and DHEA-S are important precursors for the production of estrogen and androgens, DHEA treatment may be a physiological strategy to reduce the symptoms of hormone deficiency in postmenopausal women (Davis et al., 2011). Another study reported that TC and DHEA-S declined steadily between the ages of 20 and 45 years, which was associated with a slight change in TC but a persistent decrease in DHEA-S in menopause. An age-related decline in DHEA-S level in women with polycystic ovary syndrome (PCOS) was also shown (Schmidt et al., 2011). PRL is also an important stress-induced hormone that increases in postmenopausal women who suffer from acute illness. A study with a specific focus on postmenopausal women showed no significant difference between postmenopausal women and the control group who were healthy postmenopausal women in the PRL levels (Raj et al., 2016). Based on our knowledge, no studies have been conducted on PRL to DHEA-S ratio. In the present study, the decrease in the amount of both hormones after treatment with herbal medicine was only significant in within-group comparisons but not between-group comparisons. The increase in PRL to DHEA-S ratio in both study groups was not significant at the end of the trial in any of the within-) or between-group comparisons (Table 3). In our previous study, i.e., the study by Emaminia et al. (2020), we showed that *E. angustifolia* consumption increased testosterone levels in postmenopausal women, though this increase was not significant (Emaminia et al., 2020). Since postmenopausal women in this study did not suffer from certain stress or acute illness (Table 2), the results obtained in this study are not unexpected. It should also be noted that study participants did not report any change in their sexual desire (unpublished data) at the end of the trial in the questionnaire.

The reduction in PRL/cortisol ratio was significant in both study groups only in within-group but not between-group analyses (Table 3). However, considering the increased PRL/cortisol ratio in some autoimmune diseases (Legakis et al., 2001; Zoli et al., 2002; Koeller et al., 2004), it can be concluded that the observed decrease in PRL/cortisol ratio seems to favorably reduce the likelihood of autoimmune diseases in our study’s participants.

The DHEA-S/TSH ratio decreased after both herbal medicine and placebo treatment which was significant in within-group but not between-group comparisons (Table 3). Studies have shown that DHEA-S does not decrease uniformly with age but rather increases before menopause (Crawford et al., 2009). Tagawa et al. (2000) showed that serum concentrations of DHEA-S decreased in hypothyroidism but increased in hyperthyroidism. It was suggested thyroid hormones regulate the serum concentration of DHEA and DHEA-S. In patients with thyroid dysfunction, serum concentration of cholesterol also changes significantly; that is to say, hypercholesterolemia is associated with hypothyroidism and hypocholesterolemia is associated with hyperthyroidism (Reviewed in Tagawa et al., 2000). DHEA and thyroid hormones were not measured in our study, and the decrease in DHEA-S and increase in TSH were significant only in within-group comparisons (Table 3). In another study, we showed that 10 weeks of treatment with *E. angustifolia* did not change the levels of TC and TG but significantly decreased LDL-C and increased insulin levels. However, the insulin level remained in the normal range (Shabani et al., 2020). Thus, the increase in insulin levels after treatment with herbal medicine is probably unrelated to DHEA and TSH and is probably more affected by cortisol, which decreases glucose consumption and insulin sensitivity (Markianos et al., 1996). To our knowledge, the ratio of DHEA-S to TSH has not been studied in postmenopausal women or other study groups. Considering the changes in the lipid profile after *E. angustifolia* consumption (Shabani et al., 2020), it can be concluded that the small changes in TSH and DHEA-S levels observed in the current study are not indicative of hypothyroidism or thyroid dysfunction in postmenopausal participants However, measurement of thyroid hormones and TRH is required to understand the exact mechanism. Moreover, the significance and relevance of these changes to carbohydrate and fat metabolism need further investigation.

In both within-and between-group comparisons, the treatment with neither *E. angustifolia* nor placebo affected cortisol/TSH ratio (Table 3). However, this ratio was significantly higher in the placebo group than the herbal medicine group at the beginning of the trial (between-group *p*-value = 0.046). In the present study, the increase in cortisol after 10 weeks of treatment was significant only in the herbal medicine group and not in the between-group comparison (Table 3). As we know, the ratio of cortisol to TSH has not been investigated so far. Given the role of cortisol in stress and the role that both hormones play in regulating carbohydrate and lipid metabolism, it can be said that postmenopausal women’s metabolic status did not change significantly in terms of this hormonal ratio.

## Conclusion

The current trial was the continuation of our studies previously conducted and the first ethnopharmacological study on the effect of *E. angustifolia* on the profile of TSH, DHEA-S, PRL, cortisol, and their ratios in the postmenopausal women. The observed outcomes about the effect of *E. angustifolia* on menopause were not completely in line with the Iranian folklore. The ten-week consumption of *E. angustifolia* had no significant effects on the level of the studied hormones or their ratios. A larger sample size study on premenopausal and menopausal participants in the long term is necessary to evaluate the effect of *E. angustifolia* on menopausal symptoms in terms of the studied hormone.

### Sponsor (s)

1- Damghan University, Damghan, Iran. 2- Alborz University of Medical Sciences, Karaj, Iran.

## Data Availability

The data analyzed in this study is subject to the following licenses/restrictions: Whenever is necessary, we could provide our raw data in excel format without personal information for the participants. Requests to access these datasets should be directed to arezaei@du.ac.ir.
